# Retraction Note: Severe Viperidae envenomation complicated by a state of shock, acute kidney injury, and gangrene presenting late at the emergency department: a case report

**DOI:** 10.1186/s12873-020-00318-x

**Published:** 2020-03-30

**Authors:** Agnès Esiéné, Paul Owono Etoundi, Joel Noutakdie Tochie, Arlette Junette Mbengono Metogo, Jacqueline Ze Minkande

**Affiliations:** 1 Department of Emergency medicine, Anesthesiology and critical care, Faculty of Medicine and Biomedical Sciences, University of Yaoundé I, Yaoundé, Cameroon; 2 Department of Emergency medicine, Anesthesiology and critical care, Yaounde Central Hospital, Yaoundé, Cameroon; 3 Department of Emergency medicine, Anesthesiology and critical care, Yaoundé Gynaeco-Obstetrics and Paediatric Hospital, Yaoundé, Cameroon

**Correction to: BMC Emerg Med (2019) 19: 26**

**https://doi.org/10.1186/s12873-019-0239-0**

The authors have retracted this case report [1] because the head of the snake shown in Figure 1 and described as being that of a viper (*Echis occellatus*) is identical to the head of a snake shown in Figure 1 of a different case report [2] where it was identified as being *Naja melanoleuca*, a member of the *Elapidae* family. The authors do not have records of the manufacturer and batch numbers of the 3 vials of anti-venom serum administered to the patient in [1]. All authors agree with this retraction.
